# Trehalose protects against cadmium-induced cytotoxicity in primary rat proximal tubular cells via inhibiting apoptosis and restoring autophagic flux

**DOI:** 10.1038/cddis.2017.475

**Published:** 2017-10-12

**Authors:** Xin-Yu Wang, Heng Yang, Min-Ge Wang, Du-Bao Yang, Zhen-Yong Wang, Lin Wang

**Affiliations:** 1 College of Animal Science and Veterinary Medicine, Shandong Agricultural University, 61 Daizong Street, Tai’an City 271018, China; 2 Shandong Provincial Key Laboratory of Animal Biotechnology and Disease Control and Prevention, Shandong Agricultural University, 61 Daizong Street, Tai’an City 271018, China; 3 Shandong Provincial Engineering Technology Research Center of Animal Disease Control and Prevention, Shandong Agricultural University, 61 Daizong Street, Tai’an City 271018, China

## Abstract

Autophagy has an important renoprotective function and we recently found that autophagy inhibition is involved in cadmium (Cd)-induced nephrotoxicity. Here, we aimed to investigate the protective effect of trehalose (Tre), a novel autophagy activator, against Cd-induced cytotoxicity in primary rat proximal tubular (rPT) cells. First, data showed that Tre treatment significantly decreased Cd-induced apoptotic cell death of rPT cells via inhibiting caspase-dependent apoptotic pathway, evidenced by morphological analysis, flow cytometric and immunoblot assays. Also, administration with Tre protected rPT cells against Cd-induced lipid peroxidation. Inhibition of autophagic flux in Cd-exposed rPT cells was markedly restored by Tre administration, demonstrated by immunoblot analysis of autophagy marker proteins and GFP and RFP tandemly tagged LC3 method. Resultantly, Cd-induced autophagosome accumulation was obviously alleviated by Tre treatment. Meanwhile, blockage of autophagosome–lysosome fusion by Cd exposure was noticeably restored by Tre, which promoted the autophagic degradation in Cd-exposed rPT cells. Moreover, Tre treatment markedly recovered Cd-induced lysosomal alkalinization and impairment of lysosomal degradation capacity in rPT cells, demonstrating that Tre has the ability to restore Cd-impaired lysosomal function. Collectively, these findings demonstrate that Tre treatment alleviates Cd-induced cytotoxicity in rPT cells by inhibiting apoptosis and restoring autophagic flux.

Cadmium (Cd) is a widespread environmental toxicant of increasing importance because of its extensive use in various anthropogenic and industrial activities.^1^ It is absorbed in significant quantities from cigarette smoke, food, water and air contamination and is known to have numerous undesirable effects on both humans and animals.^2^ As a nonessential element, it exerts toxic effects on multiple organs in mammals and has been classified as a human carcinogen by the International Agency for Research on Cancer.^3^ It is now well accepted that Cd can accumulate in many organs, including liver, kidney, pancreas and testis, and adversely affect the functions of these organs.^4, 5, 6, 7^ Kidney is a major site for Cd accumulation and the primary target organ of following acute or chronic Cd exposure.^8^ The kidney proximal tubule is a major damage site of Cd nephrotoxicity.^9^ Hereby, primary rat proximal tubular (rPT) cells were established to elucidate the intracellular levels in this study.

We previously demonstrated that apoptotic death promoted by oxidative stress is the major cell death mechanism of low-level Cd-induced nephrotoxicity in rPT cells.^10^ Autophagy is an adaptive response to extracellular and intracellular stress, which is widely accepted as a cytoprotective mechanism to promote cell survival and restore cell homeostasis.^11, 12, 13^ However, our research group recently found that Cd exposure inhibits the autophagic flux in rPT cells, which has a negative impact on Cd nephrotoxicity.^14, 15^ Likewise, Cd-induced autophagy inhibition is intimately related to oxidative stress.^14, 16^ Given these obtained results, we speculated that a potent antioxidant agent with antiapoptotic and autophagy-enhancing effects might be useful in the treatment of Cd nephrotoxicity.

Trehalose (Tre), a natural occurring-linked disaccharide widely distributed in non-mammalian species such as fungi, yeast, invertebrates, insects and plants, functions to provide energy sources and protects the integrity of cells against various environmental stresses.^17^ Several studies have reported that Tre acts as an antioxidant, which has been proved to be effective against lipid peroxidation.^18, 19, 20, 21, 22, 23^ Furthermore, Tre is a novel mTOR-independent autophagy enhancer. It can activate autophagic flux and prevent the formation of cytoplasmic protein aggregation in cultured cells.^24^ Tre has also been demonstrated to protect against apoptosis in an autophagy-dependent manner.^25, 26^ Despite data that confirmed these properties of Tre, few studies have investigated the protective effect of Tre on Cd-induced nephrotoxicity till now. Hereby, this study was designed to assess whether Tre administration has a protective effect against Cd-induced nephrotoxicity *in vitro*. These results would provide a protective means against environmental Cd-induced renal damage and generate more comprehensive and reliable data for toxicological risk evaluation.

## Results

### Protective effect of Tre on Cd-induced cellular death in rPT cells

**Figure 1:**
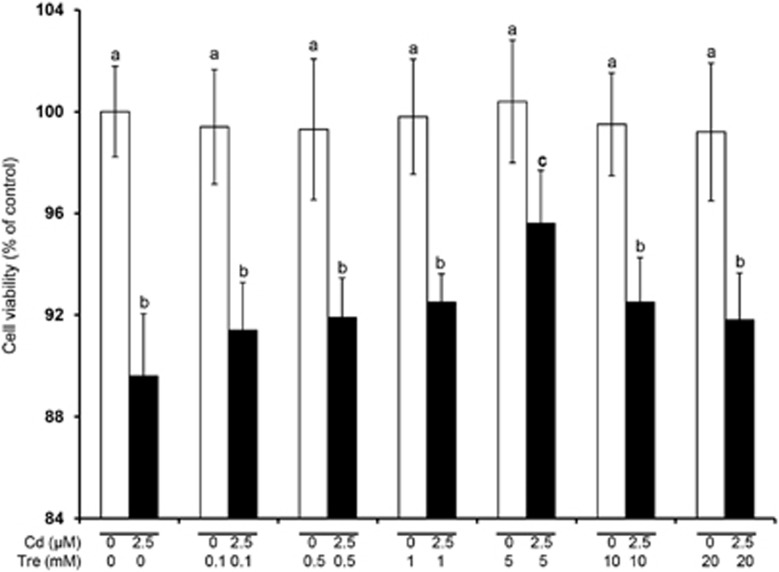
Effects of Cd and/or Tre on cell viabilities in rPT cells after 12 h treatment. Cells were incubated with a range of Tre concentrations (0, 0.1, 0.5, 1, 5, 10 and 20 mM) and/or 2.5 *μ*M Cd for 12 h to determine the cell survival. These two diverse colors were represented to point out which cells were treated with 2.5 *μ*M CdAc_2_ (black) and which were not (white). Bars with different superscripts are statistically different (*P*<0.05)

**Figure 2:**
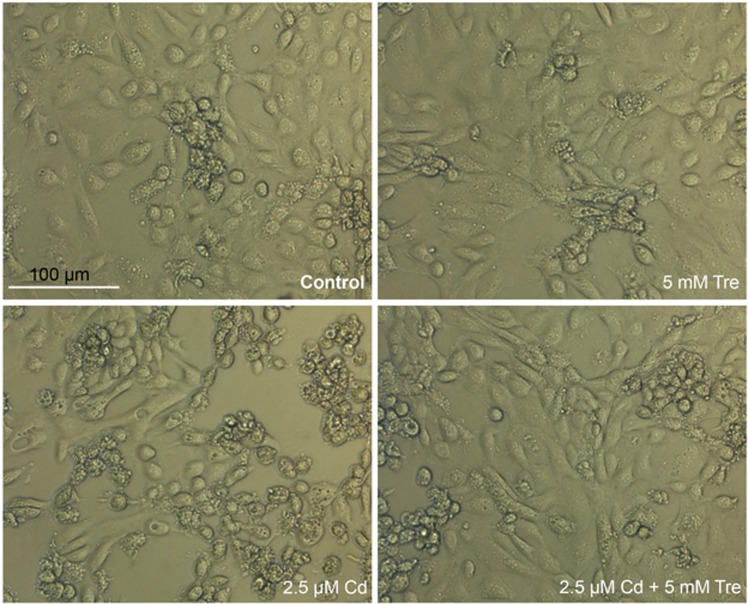
Effects of Cd and/or Tre on morphological changes in rPT cells observed under phase contrast microscope. Representative images, scale bar: 100 *μ*m

Compared with the control group, cell viabilities showed no significant changes after treating with 0.1–20 mM Tre alone for 12 h (*P*>0.05), suggesting non-toxic effect of Tre at these doses (Figure 1). Incubation of rPT cells with 5 mM Tre significantly inhibited 2.5 *μ*M Cd-induced cell death (*P*<0.05). Thus, 5 mM Tre was chosen to evaluate its protective effect on Cd-induced cytotoxicity. As shown in Figure 2, morphological analysis by phase contrast microscopy showed decreased cell density, cellular detachment, shrunk and round morphology in Cd-treated cells. However, such morphologic changes were significantly improved in 2.5 *μ*M Cd plus 5 mM Tre group, demonstrating the significant protective effect of Tre on Cd-induced cytotoxicity in rPT cells.

### Cd-induced apoptosis is reversed by Tre in rPT cells

**Figure 3:**
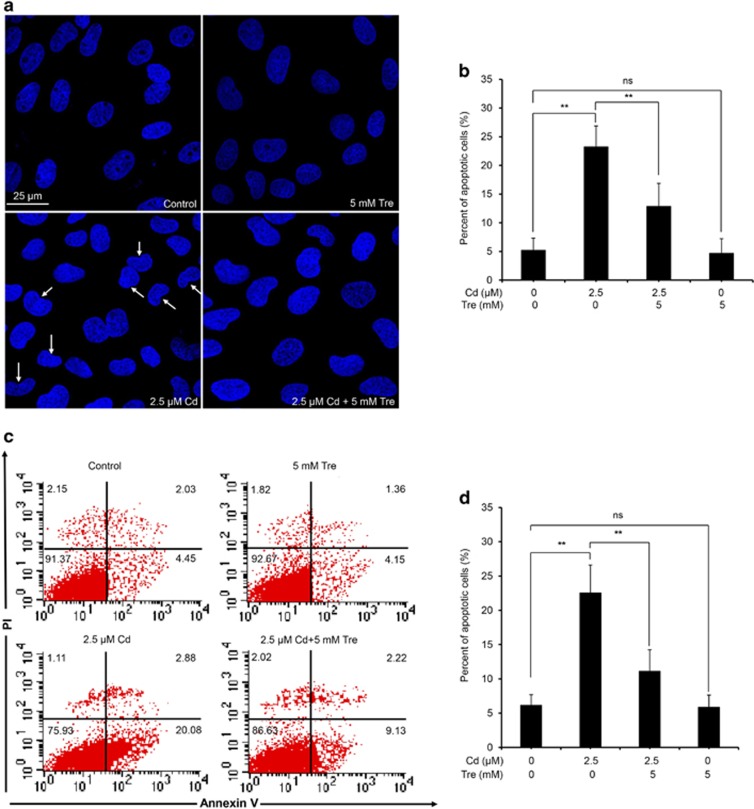
Effect of Tre on Cd-induced apoptosis in rPT cells. (**a** and **b**) Cells grown on coverslips were coincubated with 2.5 *μ*M Cd and 5 mM Tre for 12 h, and nuclear chromatin changes (apoptosis) were assessed by DAPI staining. Changes of nuclei fragmentation with condensed chromatin are evident (thin arrows). Representative morphological changes of apoptosis are present in (**a**), and its statistical result of apoptotic rates (**b**) are expressed as mean±S.E.M. (*n*=9). (**c** and **d**) Cells were treated with Cd and/or Tre for 12 h to assess the apoptosis using flow cytometry. Representative dot plots of Annexin V-PI staining are present in (**c**), and its statistical result of apoptotic rates (**d**) are expressed as mean±S.E.M. (*n*=9). NS, not significant; **P<0.01

**Figure 4:**
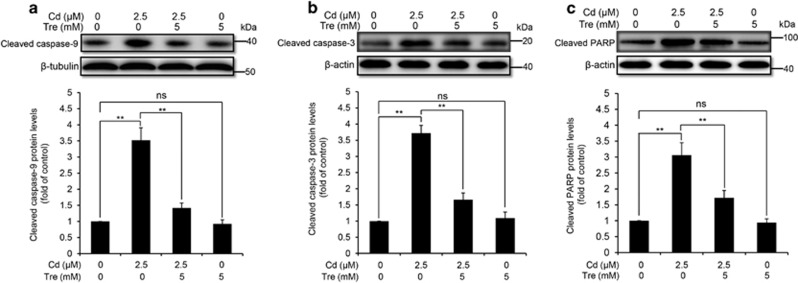
Effects of Cd and/or Tre on protein levels of apoptotic markers in rPT cells. Cells were treated with 2.5 *μ*M Cd and/or 5 mM Tre for 12 h to analyze the protein levels of cleaved caspase-9 (**a**), cleaved caspase-3 (**b**) and cleaved PARP (**c**) using western blot analysis. Upper panel representative western blot image; lower panel quantitative analysis (mean±S.E.M., *n*=4). NS, not significant; **P<0.01

It has been demonstrated that the apoptotic mechanism has a chief role in Cd-induced cellular death in rPT cells.^10^ Thus, we next determined whether Tre-mediated cytoprotective effect via inhibiting the apoptosis. First, elevation of apoptosis induced by Cd can be significantly inhibited by coincubation with Tre, measured by two methods (Figure 3). Consistent with this result, we found that Tre significantly prevented Cd-induced activation of apoptotic markers, that is, cleaved caspase-9, cleaved caspase-3 and cleaved PARP (Figure 4). However, Tre treatment alone had no effect on apoptosis and protein levels of three apoptotic markers. These data verified that Tre inhibited Cd-induced apoptosis via blocking caspase-dependent apoptotic pathway.

### Tre suppresses ROS generation and MDA level in Cd-exposed rPT cells

**Figure 5:**
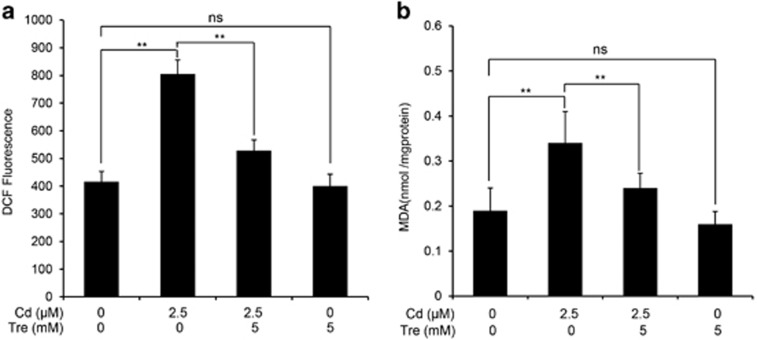
Effects of Cd and/or Tre on generation of ROS and intracellular MDA levels in rPT cells. Cells were treated with 2.5 *μ*M Cd and/or 5 mM Tre for 12 h. Then, the cells were collected. (**a**) The harvested cells were incubated with 100 *μ*M DCFH-DA for 30 min at 37 °C. DCF fluorescence was measured using flow cytometer with FL-1 filter. Fluorescence results were expressed as mean fluorescence. Each bar represents mean±S.E.M. (*n*=6). (**b**) The harvested cells were used to detect the MDA levels using a commercial kit. Data are expressed as mean±S.E.M. (*n*=6). NS, not significant; **P<0.01

The generation of intracellular reactive oxygen species (ROS) level was detected by using the fluorescent probe DCFH-DA. The intracellular ROS production with exposure to 2.5 *μ*M Cd alone for 12 h treatment was elevated (Figure 5a), whereas cotreatment with 5 mM Tre significantly (*P*<0.01) reduced the intracellular ROS level. Simultaneously, intracellular malondialdehyde (MDA) level (a common end product of lipid peroxidation) was measured to study the protective effect of Tre on Cd-induced oxidative lesions (Figure 5b). Cd exposure caused a significant generation of MDA level in rPT cells, compared with the control group, while addition of Tre significantly decreased the Cd-induced intracellular MDA level (*P*<0.01). Data in Figure 5 clearly indicated that Tre markedly alleviated Cd-induced oxidative stress in rPT cells.

### Tre restores the autophagic flux inhibited by Cd in rPT cells

**Figure 6:**
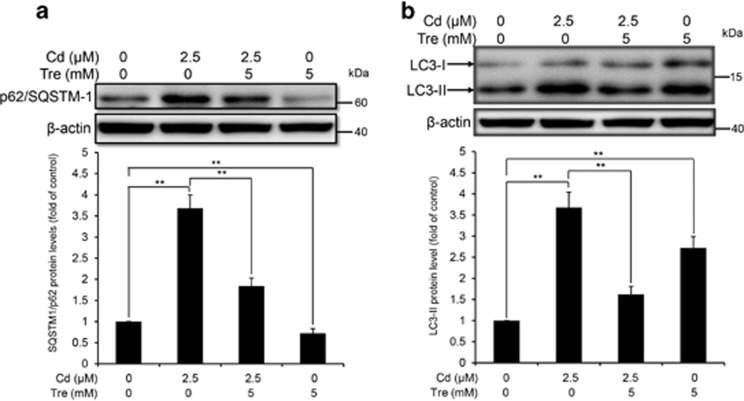
Effects of Cd and/or Tre on protein levels of p62 and LC3 in rPT cells. Cells were treated with 2.5 *μ*M Cd and/or 5 mM Tre for 12 h to assess the protein levels of p62 (**a**) and LC3 (**b**). Upper panel representative western blot image; lower panel quantitative analysis (mean±S.E.M., *n*=4). **P<0.01

**Figure 7:**
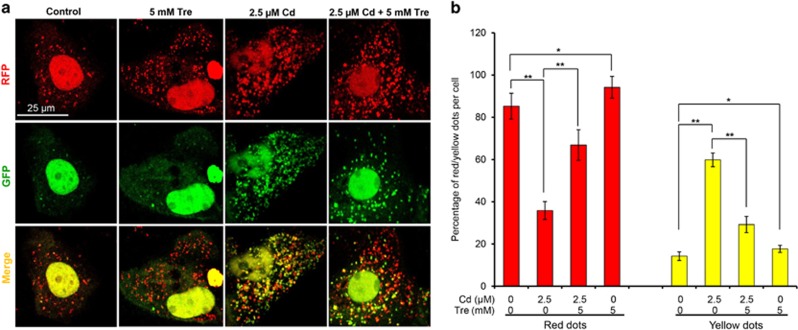
Effects of Cd and/or Tre on autophagic flux in rPT cells. Cells grown on coverslips were transfected with RFP-GFP-LC3 plasmid for 36 h, and then treated with 2.5 *μ*M Cd and/or 5 mM Tre for 12 h. (**a**) Representative confocal images of different treatments as indicated. (**b**) The number of yellow puncta (autophagosomes) and the number of red puncta (autolysosomes) in the merged images were counted and the total number of puncta per cell was calculated as percentage. Data are presented as mean±S.E.M., *n*=3 independent experiments; *P<0.05; **P<0.01

Tre has been characterized as an effective autophagy inducer in various mammalian cells.^17^ Our previous study demonstrated that Cd treatment inhibited the autophagic flux in rPT cells.^14^ Thus, we designed this study to investigate whether Tre can restore the blockade of autophagic flux in Cd-exposed rPT cells. First, immunoblot analysis of autophagy marker protein p62 and LC3 were applied to monitor the changes in autophagic flux. As shown in Figures 6, 5 mM Tre treatment alone significantly reduced p62 protein level and increased LC3-II protein level, suggesting that it is a potent autophagy enhancer. Cd exposure markedly enhanced the protein levels of p62 and LC3-II, indicating the inhibition of autophagic flux in rPT cells. However, Cd-induced accumulation of p62 and LC3-II was significantly alleviated by Tre treatment. Simultaneously, we used a valuable tool for examining autophagy flux, the tandem RFP-GFP-LC3 construct. In normal condition, the LC3-II-positive autophagosomes were labeled with yellow (GFP and RFP signals), and after fusion with lysosomes, autolysosomes were shown as red-only puncta (GFP is more rapidly quenched than RFP by low lysosomal pH).^27^ Compared with the control group, Tre treatment alone markedly induced the increase of both yellow and red-only puncta, demonstrating its role in autophagy promotion. Yellow puncta were increased with a concomitant decrease in red puncta in Cd-exposed cells, suggesting that autophagosome maturation into autolysosomes is blocked, that is, inhibition of autophagic flux (Figure 7). Meanwhile, Cd-induced increase in yellow puncta and decrease in red dots was significantly attenuated by cotreatment with 5 mM Tre. Collectively, these data demonstrate that Cd-inhibited autophagic flux can be effectively restored by administration of 5 mM Tre.

### Cd-mediated accumulation of autophagosomes is alleviated by Tre treatment

**Figure 8:**
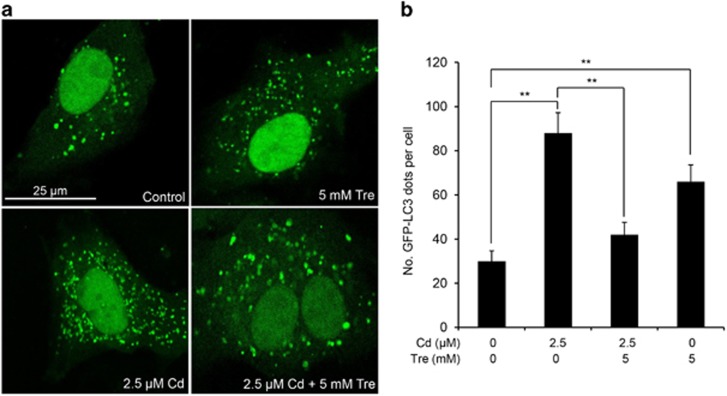
Tre alleviated Cd-induced autophagosome accumulation in rPT cells. Cells grown on coverslips were transfected with GFP-LC3 plasmid for 36 h, and then treated with 2.5 *μ*M Cd and/or 5 mM Tre for 12 h. The formation of GFP-LC3 puncta was observed under the confocal microscope and the number of GFP-LC3 puncta per cell was quantified. (**a**) Representative photomicrographs of confocal microscopy. (**b**) Quantitation of GFP-LC3 puncta was performed by counting 50 cells per condition from three independent experiments, and average numbers of puncta per cell are shown (mean±S.E.M.). **P<0.01

There was an increase in the number of autophagosomes in case of the blockage of autophagic flux; moreover, the monitoring of GFP-LC3 puncta in GFP-LC3 transfection cells is a reliable method to assess the accumulation of autophagosomes.^27^ As shown in Figure 8, a significantly increased number of GFP-LC3 puncta was present in Cd-treated cells, demonstrating that Cd-induced blockage of autophagic flux led to the accumulation of autophagosomes. However, Cd-induced autophagosome accumulation was noticeably alleviated by Tre treatment. Moreover, increased numbers of autophagosomes can be associated either with increased autophagosomes synthesis or decreased autophagosomes turnover.^27^ Thus, Tre-induced autophagosome accumulation in rPT cells may be due to its elevated autophagosome formation.

### Cd-blocked autophagosome–lysosome fusion is relieved by Tre treatment

**Figure 9:**
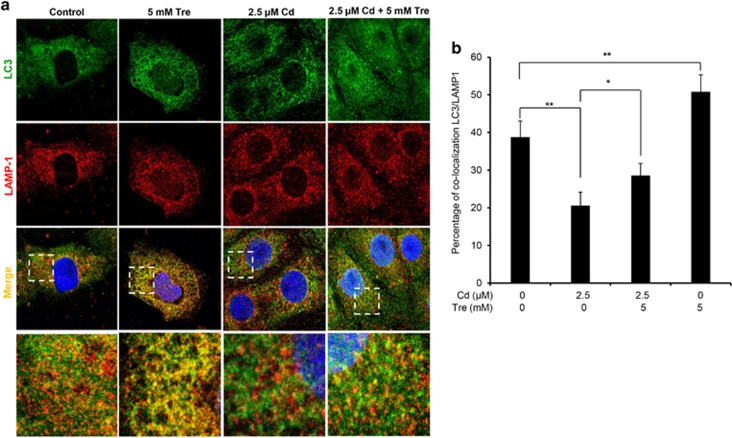
Tre restored Cd-inhibited autophagosome–lysosome fusion in rPT cells. Cells grown on coverslips were treated with 2.5 *μ*M Cd and/or 5 mM Tre for 12 h, and then successively stained with LC3 (green), LAMP-1 (red) and DAPI (blue). Colocalization of LC3 and LAMP-1 was assessed by confocal microscopy. (**A**) Representative confocal images showing colocalization of LC3 with LAMP-1. Higher magnification images of the outlined area are shown on the bottom. (**b**) Percent of colocalization of LC3 with LAMP-1. Data are represented as mean±S.E.M. of three independent experiments with 50 cells per condition in each experiment. *P<0.05; **P<0.01

Autophagy is a dynamic process during which isolation membranes package substrates to form autophagosomes that are fused with lysosomes to form autolysosomes for degrading internalized cargo.^28^ Thus, autophagosome–lysosome fusion is an essential process to maintain functional autophagic flux.^29^ LC3 is a marker protein located on the autophagosomal membrane,^30^ and LAMP-1 is known to be one of the major protein components of the lysosomal membrane.^31^ Thus, the location of autophagosome and lysosome was detected by LC3 and LAMP-1 immunostaining, respectively. By double immunofluorescence staining (colocalization analysis) (Figure 9), we confirmed that blockage of autophagosome–lysosome fusion occurred in Cd-exposed rPT cells. Treatment with 5 mM Tre alone promoted fusion of autophagosomes to lysosomes in rPT cells, while cotreatment with Tre can significantly relieve the fusion blockage of autophagosomes and lysosomes induced by Cd treatment.

### Tre contributes to reacidification of alkalinized lysosomes in Cd-exposed rPT cells

**Figure 10:**
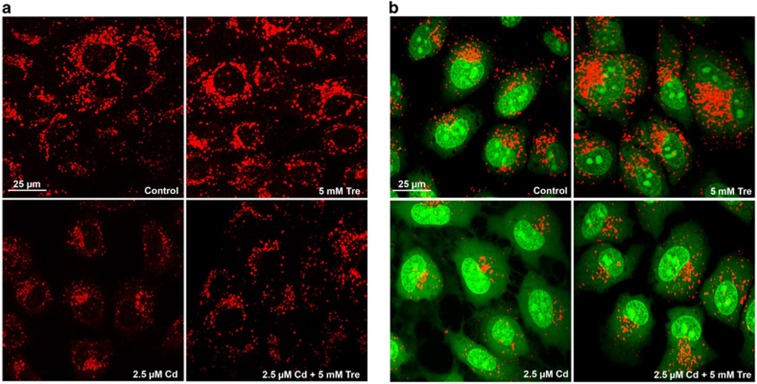
Effects of Cd and/or Tre on lysosomal acidity in rPT cells. Cells grown on coverslips were treated with 2.5 *μ*M Cd and/or 5 mM Tre, followed by staining with 100 nM LTR (**a**) or 5 *μ*g/ml AO (**b**) at 37 °C for 30 min. Slides were viewed using a scanning confocal microscope and representative confocal images were shown

It is noteworthy that impairment of lysosomal function acts as a key event to connect the blockade of autophagy flux.^32^ We then assessed whether the protective effect of Tre against Cd-induced autophagic flux was related to restoration of lysosomal function. Two sensitive lysosomotropic pH probes were applied to monitor changes in lysosomal pH in rPT cells (Figure 10). First, LysoTracker Red (LTR) manifests red fluorescence in a pH-dependent manner in the lysosome and the increased staining indicates the reduced lysosomal pH.^33^ As shown in Figure 10a, Tre treatment alone enhanced lysosomal acidification (increased LTR staining), while single Cd treatment significantly increased the lysosomal pH (decreased red fluorescence of LTR) compared with the control cells. Meanwhile, Cd-induced lysosomal alkalinization was partly reversed by cotreatment with Tre via its reacidification ability. Second, AO staining presents green fluorescence in the cytosol, but red fluorescence when it is accumulated in the acidic compartments owing to it being highly protonated.^33^ Thus, decrease in granular red fluorescence with increase in diffuse green fluorescence implies the elevated lysosomal pH. Data in Figure 10b further verified that elevation of lysosomal pH in Cd-exposed rPT cells can be effectively reacidated by adding Tre.

### Cd-impaired lysosomal degradation capacity is partly recovered by Tre treatment

**Figure 11:**
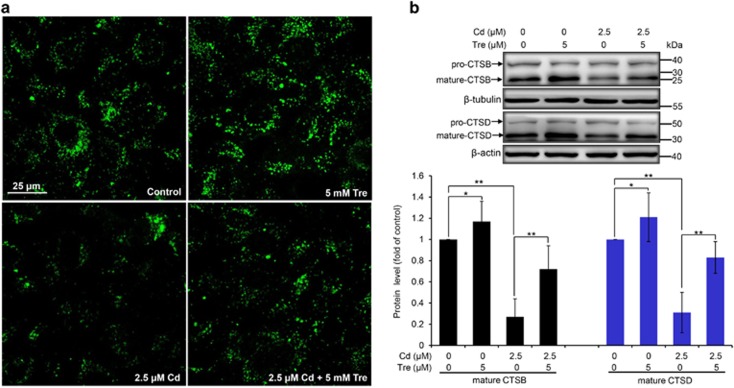
Effects of Cd and/or Tre on lysosomal proteolytic activity in rPT cells. (**a**) Cells grown on coverslips were preincubated with 10 *μ*g/ml DQ-BSA green for 12 h, and then refreshed the medium and treated with 2.5 *μ*M Cd and/or 5 mM Tre for 12 h to evaluate the lysosomal degradation capacity using confocal microscopy. (**b**) Cells were treated with 2.5 *μ*M Cd and/or 5 mM Tre for 12 h, and then protein levels of CTSB and CTSD were assessed by western blot analysis. Upper panel representative western blot image; lower panel quantitative analysis of protein levels (mean±S.E.M., *n*=4). *P<0.05; **P<0.01

Lysosomes are critical for protein degradation in the endocytic pathway and its degradation capacity is required to maintain the autophagic flux.^34^ Next, we used two methods to analyze the lysosomal degradation capacity in this study. First, DQ-BSA dequenching analysis, a lysosome-specific degradation assay, was chosen for the visualization of lysosomal proteolytic activity.^35^ When DQ-BSA is degraded under normal lysosomal conditions, it releases green fluorescence in the lysosome. As shown in Figure 11a, DQ-BSA was efficiently cleaved during Tre treatment alone, while Cd exposure caused a significant reduction of DQ-BSA-related green fluorescence, confirming that Cd inhibited lysosomal degradation capacity of rPT cells. Likewise, cotreatment with Tre restored the decreased DQ-BSA green fluorescence in Cd-exposed rPT cells. Second, cathepsin B (CTSB) and cathepsin D (CTSD) are the most abundant lysosomal proteases.^36^ Thus, intracellular protein levels of CTSB and CTSD in cells treated with Cd and/or Tre were assessed by western blotting analysis (Figure 11b). Consistently, Cd exposure impaired the maturation of CTSB and CTSD in rPT cells, which was significantly restored by the addition of Tre. Taken together, our data demonstrated that Tre has the ability to restore Cd-inhibited lysosomal degradation capacity in rPT cells.

## Discussion

In response to numerous stresses, autophagy is a mechanism to maintain intracellular homeostasis with cytoprotective effects by eliminating and recycling of damaged macromolecules and organelles.^37^ Evidence from numerous recent studies corroborate that autophagy has an important renoprotective function in tubular epithelial cells.^15, 38, 39, 40, 41^ Our research group recently demonstrated that blockade of autophagic flux contributes to Cd-induced cytotoxicity in rPT cells,^14, 15^ which gives us a hint that a potent autophagy activator may help to alleviate Cd-induced nephrotoxicity. Tre has been described as an autophagy enhancer with antioxidative ability, but there is a lack of investigation on the protective effect of Tre against Cd-induced nephrotoxicity. Herein, the present experiments provide the first insight into the cytoprotective mechanism of Tre against Cd-induced nephrotoxicity *in vitro* via attenuating apoptosis and restoring autophagic flux.

Tre is a non-toxic naturally occurring disaccharide that can be administered safely and orally and has been accepted as a safe food ingredient by the European regulation system following approval by the US Food and Drug Administration.^20, 42^ Data in Figure 1 verified that Tre is non-toxic to rPT cells. Recent studies have demonstrated that Tre was an effective cryoprotective reagent through preventing apoptosis.^21, 23, 25, 26^ It was also proved that Tre-based eye drops is effective in the treatment of severe human dry eye through the suppression of apoptosis.^43^ Consistent with these previous results, our data (Figures 1, 2, 3, 4) corroborate the protective effect of Tre against Cd-induced apoptotic death by inhibiting caspase-dependent pathway; however, whether other apoptotic pathways have a part in this process remains to be further clarified.

There is general consensus that oxidative stress contributes to the development of Cd nephrotoxicity. Moreover, oxidative stress has a critical role in the apoptosis of rPT cells during Cd exposure.^10, 44^ In lower organisms, as well as experimentally in mammals, Tre has been proved to be effective against oxidative stress.^18, 19, 21, 22^ Consistent with previous reports, Tre administration greatly alleviated Cd-induced intracellular ROS production and MDA levels (markers of oxidative stress) in rPT cells (Figure 5). On that basis, we considered that the antioxidant activity of Tre might be responsible for its antiapoptosis effect against Cd-induced cytotoxicity in rPT cells. Furthermore, mounting evidence suggests that oxidative stress and autophagy are intimately connected in kidney health and disease.^37^ We also demonstrated that oxidative stress is involved in Cd-induced autophagy inhibition in rPT cells.^15^

As Tre possesses the antioxidative and autophagy-enhancing effects, we next investigate the potential role of Tre administration in the course of Cd-induced autophagy status in rPT cells. The term 'autophagic flux' is used to denote the dynamic process of autophagosome synthesis, delivery of autophagic substrates to the lysosome and degradation of autophagic substrates inside the lysosome, and is a reliable indicator of autophagic activity.^45^ Immunoblot analysis of LC3 and p62, two autophagy marker proteins, has been widely used to monitor autophagic flux.^46^ It is a consensus that enhanced p62 protein level has been regarded as an indicator for blockage of autophagic flux,^46, 47^ and the net amount of cytosolic LC3-II is a critical hallmark for monitoring autophagy in mammalian cells.^46^ We previously proved that Cd-induced inhibition of autophagic flux results from overall autophagic degradation rather than autophagosome formation.^15^ Here, data in Figure 6 showed that Cd-induced impairment of autophagic flux was markedly restored by the addition of Tre. Simultaneously, a more sensitive and dynamic assay, that is, GFP and RFP tandemly tagged LC3 method, was applied to assess the autophagic flux.^48^ As shown in Figure 7, fluorescence color change of RFP-GFP-LC3 and quantitative analysis of autophagosomes (yellow puncta) and autolysosomes (red puncta) give us a solid conclusion that impairment of autophagic flux in Cd-exposed rPT cells was obviously relieved by Tre administration. Resultantly, Cd-induced autophagosomes accumulation was significantly alleviated by the addition of Tre due to its restoration of autophagic flux (Figure 8). It has been shown that excessive autophagosome accumulation may induce apoptotic cell death via the intrinsic apoptotic pathway,^49, 50, 51^ which accounts for the interplay between autophagy and apoptosis.

Fusion of autophagosomes with lysosomes to form autolysosome constitutes the second stage of autophagic flux, which is critical for the maturation of autolysosomes and degradation of autophagosome.^52^ Impairment of autophagosome–lysosome fusion leads to the accumulation of autophagosomes.^53^ In this study, Tre administration obviously promoted the formation of autolysosomes (Figure 7) and restored the impairment of autophagosome–lysosome fusion (Figure 9) in Cd-exposed cells, convincingly demonstrating the restoration effect of Tre on impaired autophagic flux by acting on the process of autophagosome–lysosome fusion.

Another mechanism for impairment of autophagic flux is lysosomal dysfunction. The lysosome is the only way to degrade autophagic cargos, thus defective lysosomal function can cause the impairment of autophagic flux.^15, 54, 55^ One unique feature of lysosome is its highly acidic pH (4.5–5.0) that provides an optimal condition for its hydrolytic enzymes to perform their catalytic function.^52^ Here, we attempt to determine the possibility that alleviation of Cd-induced autophagosome accumulation by Tre is related to changes in lysosomal pH. In this study, Cd-induced lysosomal alkalinization in rPT cells was obviously alleviated by cotreatment with 5 mM Tre (Figure 10), demonstrating that Tre has the potential to restore Cd-induced autophagy inhibition via regulating lysosomal pH. Furthermore, it is known that lysosomal proteolytic activity is dependent on acidic pH, which prompted us to further ascertain the role of lysosomal proteolytic capacity in the process of Tre against Cd-induced autophagy inhibition in rPT cells. We did find that Cd inhibited lysosome-specific degradation capacity of rPT cells by DQ-BSA dequenching analysis, which was effectively relieved by cotreatment with Tre (Figure 11a). Cathepsins are the major lysosomal proteases involved in autophagic degradation, wherein CTSB and CTSD are two abundant lysosomal proteases.^56, 57^ As the maturation (activation) of cathepsin proteases requires acidification, the altered pH ultimately resulted in greatly decreased protein degradation.^58^ As shown in Figure 11b, Cd impaired the maturation of CTSB and CTSD in rPT cells, which was markedly restored by cotreatment with Tre. Given these results, it can be concluded that Tre restored the impaired autophagic flux in Cd-treated rPT cells partly by enhancing lysosomal function. Moreover, the potential use of Tre in clinical treatment is an interesting issue. Mounting evidence has shown that Tre treatment has important neuroprotective effects in animal models of Alzheimer disease, amyotrophic lateral sclerosis, Parkinson disease due to its antiapoptotic effect and autophagic clearance of abnormal protein aggregates.^24, 59, 60, 61^ However, to date, Tre treatment has not entered into clinical use.

**Figure 12:**
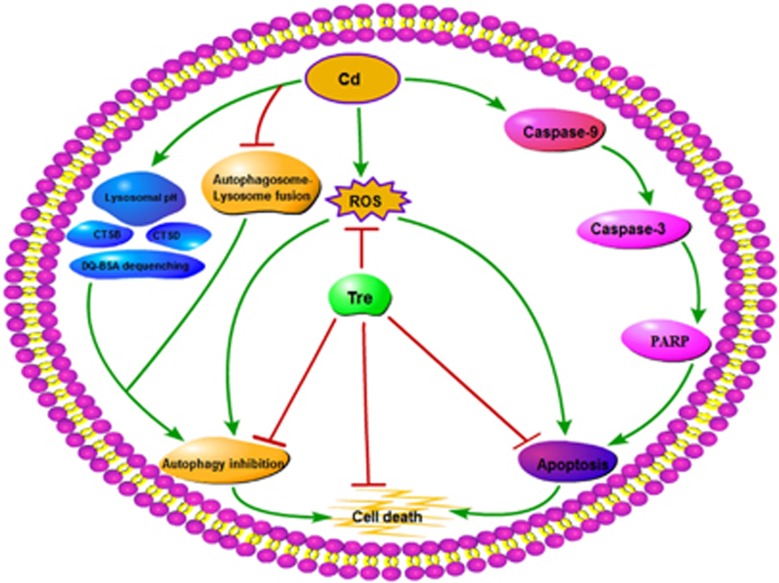
Graphical diagram representing the protective mechanism of Tre against Cd-induced cell death in rPT cells

In summary, the possible protective mechanism of Tre against Cd-induced cell death in rPT cells via inhibition of apoptosis and restoration of autophagy inhibition is expounded (Figure 12). First, Tre treatment blocks Cd-induced apoptosis by inactivating the caspase-dependent apoptotic pathway. Second, Cd-induced inhibition of autophagosome–lysosome fusion and impairment of lysosomal function (lysosomal alkalinization and decreased lysosomal degradation capacity) results in autophagy inhibition in rPT cells, which can be significantly restored by Tre administration. Particularly, Tre alleviates Cd-mediated oxidative stress, which is intimately correlated with its antiapoptosis and autophagy-enhancing effects. These findings will provide us an effective protective agent against Cd-induced nephrotoxicity.

## Materials and methods

### Chemicals and antibodies

All chemicals available were of highest grade purity. Cd acetate (CdAc_2_), d-(+)-Tre dehydrate (Tre, T0167), 4′,6-diamidine-2′-phenylindole dihydrochloride (DAPI), acridine orange (AO, A6014), propidium iodide and DMEM-F_12_ (1 : 1) medium were purchased from Sigma-Aldrich (Carlsbad, CA, USA). ROS assay kit (containing positive control and dichlorofluorescin diacetate (DCFH-DA), S0033) and Enhanced Cell Counting Kit-8 (CCK-8, C0042) were from Beyotime Biotechnology (Haimen, China). MDA Assay Kit and Annexin V Apoptosis Detection Kit were obtained from Keygen Biotech Co. Ltd (Nanjing, Jiangsu, China). LysoTracker Deep Red (LTR) (L12492), self-quenched bodipy-conjugated BSA (DQ-BSA-Green) (D-12050) and Lipofectamine 3000 Transfection Reagent (L3000015) were purchased from Invitrogen (Rockford, IL, USA). BCA Protein Assay Kit and Enhanced Chemiluminescence (ECL) Kit were obtained from Thermo Fisher Scientific Pierce (Rockford, IL, USA). The following primary antibodies were used: anti-p62/SQSTM1 (P0067), anti-LC3B (L7543), *α*-tubulin (T6199) and anti-*β*-actin (Sigma, St. Louis, MO, USA; A5441) were purchased from Sigma. Cleaved caspase-3 (9661), cleaved caspase-9 (9507) and cleaved PARP (9545) were obtained from Cell Signaling Technology (Danvers, MA, USA). LAMP-1 (sc-20011), anti-CTSB (sc-13985) and anti-CTSD (sc-6486) were from Santa Cruz Biotechnology (Santa Cruz, CA, USA). Secondary antibodies for western blotting analysis were conjugated to horseradish peroxidase (Jackson Immuno Research, West Grove, PA, USA; 705-505-303 and 111-006-062). Alexa Fluor 488-conjugated donkey anti-rabbit (ab150073) and Alexa Fluor 555-conjugated goat anti-mouse (ab150114) secondary antibodies were purchased from Abcam (Cambridge Science Park, Cambridge, UK).

### Cell isolation, culture and treatment

All procedures followed the ethics guidelines and were approved by the Animal Care and Use Committee of Shandong Agricultural University. Isolation, identification and culture of Sprague–Dawley rPT cells were as described previously.^62^ Based on the doses of Cd in our previous study,^10^ 2.5 *μ*M Cd was applied in this study. As for the optimal concentration of Tre chosen for this experiment, cells were treated with a range of Tre concentrations (0, 0.1, 0.5, 1, 5, 10 and 20 mM) and/or 2.5 *μ*M Cd for 12 h (Figure 1), and cell viabilities were tested using CCK-8 assay. The stock solution of CdAc_2_ and Tre were dissolved in sterile ultrapure water. Based on an initial screening, cell cultures undergoing exponential growth were incubated with 5 mM Tre and/or 2.5 *μ*M Cd for 12 h. After 12 h treatment, cell culture photos were taken under phase-control microscopy (Olympus, Tokyo, Japan) to check cell morphology.

### Cell viability assay (CCK-8 assay)

CCK-8 is a one-bottle solution, which contains water-soluble tetrazolium salt. It can reduce the dehydrogenase in the mitochondria to water-soluble formazan dyes. The absorbance of these formazan dyes at 450 nm is proportional to the number of viable cells in the medium. In this study, cells were seeded at a density of 1 × 10^4^ in 96-well plates. After the preprocessing, cells were treated with a series of Cd and/or Tre doses for 12 h to assess the cytoprotective effect of Tre on cell survival. After 12 h treatment, cell viability assays were performed using CCK-8, according to the manufacturer's instructions. The absorbance was read at 450 nm by the microplate reader (Sunrise, Salzburg, Austria).

### Assessment of apoptosis by morphological changes and flow cytometry

Cells were coincubated with 2.5 *μ*M Cd and/or 5 mM Tre for 12 h to assess the effect of Tre on Cd-induced apoptosis. Apoptosis is characterized morphologically by condensation and fragmentation of nuclei. Thus, DAPI staining was firstly applied to assess the morphological changes of treated cells, and 200 cells were randomly selected to count those apoptotic cells within every batch of experiment, each one performed in triplicate. Another concern is the quantitative analysis of apoptosis by flow cytometry. Both of these two methods have been extensively described in our previous study.^63^

### Measurement of lipid peroxidation biomarkers

ROS and MDA, two biomarkers of lipid peroxidation, were chosen to assess the protective effect of Tre on Cd-induced oxidative stress in rPT cells. After the designated treatments, 1.5 × 10^6^ harvested cells per ml was incubated with 100 *μ*M DCFH-DA for 30 min in dark at 37 °C. The incubated cells were harvested, suspended in PBS and ROS generation was measured by the fluorescence intensity (FL-1, 530 nm) of 10 000 cells on flow cytometer. Also, the harvested cells were lyzed in ice-cold physiological saline by sonication followed by centrifugation at 15 000 × *g* for 5 min at 4 °C. The resulting supernatants were used immediately for measuring the MDA level. The quantification of MDA was based on measuring formation of thiobarbituric acid reactive substances according to the manufacturer’s protocol. The reaction mixture was incubated at 95 °C for 40 min. After cooling, the chromogen was read spectrophotometrically at 532 nm and the MDA level was expressed in nmol/mg protein.

### Western blotting analysis

After treatment with Cd and/or Tre for 12 h, cells were collected, lysed in ice-cold RIPA buffer supplemented with protease inhibitor (Merck Millipore, Darmstadt, Germany) to prepare the total cell lysates. After protein quantification with BCA method, samples were subjected to SDS-PAGE gels and transferred to PVDF membranes. After blocking with 5% skim milk for 1 h at room temperature, membranes were incubated overnight at 4 °C with the following primary antibodies: p62 (diluted 1 : 1000), LC3B (diluted 1 : 1000), cleaved caspase-9 (diluted 1 : 1000), cleaved caspase-3 (diluted 1 : 1000), cleaved PARP (diluted 1 : 1000), CTSB (diluted 1 : 150), CTSD (diluted 1 : 150), *α*-tubulin (diluted 1 : 1000) and *β*-actin (diluted 1 :  5000). After several washes with TBST, the membranes were incubated with appropriate secondary antibodies (1 : 5000 dilution) for 50 min at room temperature. Finally, each protein was detected on a Chemidoc XRS (Bio-Rad, Marnes-La-Coquette, France) by using the ECL Kit/Thermo Fisher (Rockford, IL, USA). Proteins levels were determined by computer-assisted densitometric analysis (Densitometer, GS-800, Bio-Rad Quantity One, Marnes-La-Coquette, France). The density of each band was normalized to its respective loading control (*β*-actin or *α*-tubulin). Data obtained were expressed as the ratio of intensity of the protein in chemical-treated cells to that of the corresponding protein in control cells. Each test was performed in four experiments with different batches of cells.

### Plasmids and transient transfection

RFP-GFP-LC3 and GFP-LC3 plasmids were kind gifts of Dr. Xiao-Ming Yin (Department of Pathology and Laboratory Medicine, Indiana University School of Medicine, Indianapolis, IN, USA). Cultures of rPT cells at 60 to 80% confluence were transiently transfected with empty vector, RFP-GFP-LC3 or GFP-LC3 plasmids using Lipofectamine 3000 (Invitrogen, Carlsbad, CA, USA) according to the manufacturer's protocol. After the indicated treatments, cells were fixed with 4% paraformaldehyde for 8 min at room temperature, and then visualized by the confocal microscope (TCS SPE; Leica, Mannheim, Germany). Representative cells were selected and photographed. The number of puncta per cell was quantified using the 'analyze particles; function of ImageJ under identical threshold conditions.

### Immunofluorescence staining

Cells were seeded on sterile coverslips placed in 24-well plates. After incubating with 2.5 *μ*M Cd and/or 5 mM Tre for 12 h, cells were fixed with 4% paraformaldehyde for 8 min, permeabilized with 0.1% Triton X-100 in PBS for 15 min and blocked with 2% bovine serum albumin in PBS for 1 h at room temperature. Slides were first stained with anti-LC3 antibody (1 : 150 diluted in PBS) at 4 °C overnight. After washing the cells with PBS, cells were incubated with Alexa Fluor 488-conjugated donkey anti-rabbit secondary antibody (1 : 600 diluted in PBS) for 1 h at room temperature and washed with PBS again. Subsequently, cells were stained with anti-LAMP-1 antibody (1 : 80 diluted in PBS) at 4 °C overnight, washed with PBS again and incubated with Alexa Fluor 555-conjugated goat anti-mouse secondary antibody (1 : 500 diluted in PBS) for 1 h at room temperature. Nuclei were stained with DAPI (blue). Finally, all slides were mounted with ProLong Gold Antifade Mountant. Images were conducted on the Leica TCS SPE confocal microscope with a × 63 (1.3 numerical aperture) oil-immersion objective. Images for colocalization analysis (percentage of protein–protein colocalization) were assessed using the JaCoP plugin in ImageJ after thresholding of individual frames.^64^ All colocalization calculations were performed on three independent experiments with 50 cells per condition in each experiment. Images were prepared for presentation using Adobe Photoshop 6.0 (San Jose, CA, USA).

### AO staining and LTR staining

AO staining and LTR staining were applied to assess the functional state of lysosomes in this study. After treatment with 2.5 *μ*M Cd and/or 5 mM Tre for 12 h, cells grown on coverslips were loaded with 5 *μ*g/ml AO at 37 °C for 30 min, rinsed two times with warm (37 °C) PBS and examined under confocal laser-scanning microscope (TCS SPE; Leica, Germany) with excitation at 488 nm. Green fluorescence (emission peak between 530 and 550 nm) and red fluorescence (emission peak at about 650 nm) were simultaneously collected by two separate windows. Meanwhile, cells were incubated with 100 nM LTR (diluted in DMEM-F_12_ medium) for 30 min under ideal growth conditions (37 °C, 5% CO_2_) to label the lysosomes. Then, slides were rapidly washed with warm PBS (37 °C) for three times, mounted as described above and observed under a laser-scanning confocal microscope (TCS SPE; Leica).

### Analysis of lysosomal degradation capacity

DQ-BSA-Green was used to determine the lysosomal degradation capacity. Cells grown on coverslips in 24-well plates were incubated with 10 *μ*g/ml of DQ-BSA-Green for 12 h (37 °C, 5% CO_2_), washed two times with PBS to remove excess probe and refreshed the medium. Then, cells were treated with 2.5 *μ*M Cd and/or 5 mM Tre for 12 h. After the treatment, slides were mounted and observed under a laser-scanning confocal microscope with excitation set at 488 nm. Degradation capacity was measured by the green fluorescence signal released due to the degradation of DQ-BSA-Green.

### Data presentation

Experiments were performed at least three times with similar results. Data are presented as the mean±S.E.M. of the indicated number of replicates. Statistical comparisons were made using one-way analysis of variance (ANOVA) (Scheffe’s *F* test) after ascertaining the homogeneity of variance between the treatments, and *P*<0.05 was regarded as significant.

## References

[bib1] Thévenod F, Lee WK. Cadmium and cellular signaling cascades: interactions between cell death and survival pathways. Arch Toxicol 2013; 87: 1743–1786. 23982889 10.1007/s00204-013-1110-9

[bib2] Rani A, Kumar A, Lal A, Pant M. Cellular mechanisms of cadmium-induced toxicity: a review. Int J Environ Health Res 2014; 24: 378–399. 24117228 10.1080/09603123.2013.835032

[bib3] Waisberg M, Joseph P, Hale B, Beyersmann D. Molecular and cellular mechanisms of cadmium carcinogenesis. Toxicology 2003; 192: 95–117. 14580780 10.1016/s0300-483x(03)00305-6

[bib4] Li JL, Guo R, Li S, Wang JT, Tang ZX, Xu SW. Testicular toxicity induced by dietary cadmium in cocks and ameliorative effect by selenium. BioMetals 2010; 23: 695–705. 20372978 10.1007/s10534-010-9334-0

[bib5] Shao CC, Li N, Zhang ZW, Su J, Li S, Li JL et al. Cadmium supplement triggers endoplasmic reticulum stress response and cytotoxicity in primary chicken hepatocytes. Ecotoxicol Environ Saf 2014; 106: 109–114. 24836885 10.1016/j.ecoenv.2014.04.033

[bib6] Xu S, Pi H, Chen Y, Zhang N, Guo P, Lu Y et al. Cadmium induced Drp1-dependent mitochondrial fragmentation by disturbing calcium homeostasis in its hepatotoxicity. Cell Death Dis 2013; 4: e540. 23492771 10.1038/cddis.2013.7 PMC3615741

[bib7] Zhang F, Xing S, Li Z. Antagonistic effects of lycopene on cadmium-induced hippocampal dysfunctions in autophagy, calcium homeostatis and redox. Oncotarget 2017; 8: 44720–44731. 28615536 10.18632/oncotarget.18249 PMC5546513

[bib8] Yang H, Shu Y. Cadmium transporters in the kidney and cadmium-induced nephrotoxicity. Int J Mol Sci 2015; 16: 1484–1494. 25584611 10.3390/ijms16011484 PMC4307315

[bib9] Sabolić I, Herak-Kramberger CM, Brown D. Subchronic cadmium treatment affects the abundance and arrangement of cytoskeletal proteins in rat renal proximal tubule cells. Toxicology 2001; 165: 205–216. 11522379 10.1016/s0300-483x(01)00450-4

[bib10] Wang L, Cao J, Chen DW, Liu XZ, Lu H, Liu ZP. Role of oxidative stress, apoptosis, and intracellular homeostasis in primary cultures of rat proximal tubular cells exposed to cadmium. Biol Trace Elem Res 2009; 127: 53–68. 18802671 10.1007/s12011-008-8223-7

[bib11] Tang ZH, Zhang LL, Li T, Lu JH, Ma DL, Leung CH et al. Glycyrrhetinic acid induces cytoprotective autophagy via the inositol-requiring enzyme 1α-c-Jun N-terminal kinase cascade in non-small cell lung cancer cells. Oncotarget 2015; 6: 43911–43926. 26549806 10.18632/oncotarget.6084 PMC4791276

[bib12] Chen L, Li G, Peng F, Jie X, Dongye G, Cai K et al. The induction of autophagy against mitochondria-mediated apoptosis in lung cancer cells by a ruthenium (II) imidazole complex. Oncotarget 2016; 7: 80716–80734. 27811372 10.18632/oncotarget.13032 PMC5348350

[bib13] Yin H, Yang X, Gu W, Liu Y, Li X, Huang X et al. HMGB1-mediated autophagy attenuates gemcitabine-induced apoptosis in bladder cancer cells involving JNK and ERK activation. Oncotarget 2017; 8: 71642–71656. 29069735 10.18632/oncotarget.17796 PMC5641078

[bib14] Liu F, Li ZF, Wang ZY, Wang L. Role of subcellular calcium redistribution in regulating apoptosis and autophagy in cadmium-exposed primary rat proximal tubular cells. J Inorg Biochem 2016; 164: 99–109. 27665314 10.1016/j.jinorgbio.2016.09.005

[bib15] Liu F, Wang XY, Zhou XP, Liu ZP, Song XB, Wang ZY et al. Cadmium disrupts autophagic flux by inhibiting cytosolic Ca^2+^-dependent autophagosome–lysosome fusion in primary rat proximal tubular cells. Toxicology 2017; 383: 13–23. 28347754 10.1016/j.tox.2017.03.016

[bib16] So KY, Oh SH. Cadmium-induced heme-oxygenase-1 expression plays dual roles in autophagy and apoptosis and is regulated by both PKC-δ and PKB/Akt activation in NRK52E kidney cells. Toxicology 2016; 370: 49–59. 27658547 10.1016/j.tox.2016.09.010

[bib17] Wang QR, Ren J. mTOR-independent autophagy inducer trehalose rescues against insulin resistance-induced myocardial contractile anomalies: role of p38 MAPK and Foxo1. Pharmacol Res 2016; 111: 357–373. 27363949 10.1016/j.phrs.2016.06.024 PMC5026602

[bib18] Benaroudj N, Lee DH, Goldberg AL. Trehalose accumulation during cellular stress protects cells and cellular proteins from damage by oxygen radicals. J Biol Chem 2001; 276: 24261–24267. 11301331 10.1074/jbc.M101487200

[bib19] Tanaka M, Machida Y, Nukina N. A novel therapeutic strategy for polyglutamine diseases by stabilizing aggregation-prone proteins with small molecules. J Mol Med 2005; 83: 343–352. 15759103 10.1007/s00109-004-0632-2

[bib20] Schiraldi C, Di Lernia I, De Rosa M. Trehalose production: exploiting novel approaches. Trends Biotechnol 2002; 20: 420–425. 12220904 10.1016/s0167-7799(02)02041-3

[bib21] Liu Q, Xu L, Jiao SX, Wang TX, Song Y, Wen ZK. Trehalose inhibited the phagocytosis of refrigerated platelets *in vitro* via preventing apoptosis. Transfusion 2009; 49: 2158–2166. 19555421 10.1111/j.1537-2995.2009.02254.x

[bib22] Béranger F, Crozet C, Goldsborough A, Lehmann S. Trehalose impairs aggregation of PrPSc molecules and protects prion-infected cells against oxidative damage. Biochem Biophys Res Commun 2008; 374: 44–48. 18602368 10.1016/j.bbrc.2008.06.094

[bib23] Lu H, Zhu Z, Dong L, Jia X, Sun X, Yan L et al. Lack of trehalose accelerates H_2_O_2_-induced Candida albicans apoptosis through regulating Ca^2+^ signaling pathway and caspase activity. PLoS ONE 2011; 61: e15808. 10.1371/journal.pone.0015808 PMC3016397 21246042

[bib24] Tanji K, Miki Y, Maruyama A, Mimura J, Matsumiya T, Mori F et al. Trehalose intake induces chaperone molecules along with autophagy in a mouse model of Lewy body disease. Biochem Biophys Res Commun 2015; 465: 746–752. 26299928 10.1016/j.bbrc.2015.08.076

[bib25] Chen W, Zhang X, Liu M, Zhang J, Ye Y, Lin Y et al. Trehalose protects against ocular surface disorders in experimental murine dry eye through suppression of apoptosis. Exp Eye Res 2009; 89: 311–318. 19345212 10.1016/j.exer.2009.03.015

[bib26] Honma Y, Sato-Morita M, Katsuki Y, Mihara H, Baba R, Harada M. Trehalose activates autophagy and decreases proteasome inhibitor-induced endoplasmic reticulum stress and oxidative stress-mediated cytotoxicity in hepatocytes. Hepatol Res 2017 (doi:10.1111/hepr.12892). 10.1111/hepr.12892 28295916

[bib27] Mizushima N, Yoshimori T, Levine B. Methods in mammalian autophagy research. Cell 2010; 140: 313–326. 20144757 10.1016/j.cell.2010.01.028 PMC2852113

[bib28] Lu Y, Dong S, Hao B, Li C, Zhu K, Guo W et al. Vacuolin-1 potently and reversibly inhibits autophagosome–lysosome fusion by activating RAB5A. Autophagy 2014; 10: 1895–1905. 25483964 10.4161/auto.32200 PMC4502727

[bib29] Mauvezin C, Neufeld TP. Bafilomycin A1 disrupts autophagic flux by inhibiting both V-ATPase-dependent acidification and Ca-P60A/SERCA-dependent autophagosome–lysosome fusion. Autophagy 2015; 11: 1437–1438. 26156798 10.1080/15548627.2015.1066957 PMC4590655

[bib30] Yu J, Lan L, Lewin SJ, Rogers SA, Roy A, Wu X et al. Identification of novel small molecule Beclin 1 mimetics activating autophagy. Oncotarget 2017; 8: 51355–51369. 28881653 10.18632/oncotarget.17977 PMC5584254

[bib31] Chen JW, Madamanchi N, Madamanchi NR, Trier TT, Keherly MJ. Lamp-1 is upregulated in human glioblastoma cell lines induced to undergo apoptosis. J Biomed Sci 2001; 8: 365–374. 11455200 10.1007/BF02258379

[bib32] Yu L, Wu WK, Gu C, Zhong D, Zhao X, Kong Y et al. Obatoclax impairs lysosomal function to block autophagy in cisplatin-sensitive and -resistant esophageal cancer cells. Oncotarget 2016; 7: 14693–14707. 26910910 10.18632/oncotarget.7492 PMC4924745

[bib33] Boya P, Kroemer G. Lysosomal membrane permeabilization in cell death. Oncogene 2008; 27: 6434–6451. 18955971 10.1038/onc.2008.310

[bib34] Mizushima N, Komatsu M. Autophagy: renovation of cells and tissues. Cell 2011; 147: 728–741. 22078875 10.1016/j.cell.2011.10.026

[bib35] Frost LS, Dhingra A, Reyes-Reveles J, Boesze-Battaglia K. The use of DQ-BSA to monitor the turnover of autophagy-associated cargo. Methods Enzymol 2017; 587: 43–54. 28253971 10.1016/bs.mie.2016.09.052 PMC5338641

[bib36] Liu S, Sarkar C, Dinizo M, Daden AI, Koh EY, Lipinski MM et al. Disrupted autophagy after spinal cord injury is associated with ER stress and neuronal cell death. Cell Death Dis 2015; 6: e1582. 25569099 10.1038/cddis.2014.527 PMC4669738

[bib37] Sureshbabu A, Ryter SW, Choi ME. Oxidative stress and autophagy: crucial modulators of kidney injury. Redox Biol 2015; 4: 208–214. 25613291 10.1016/j.redox.2015.01.001 PMC4803795

[bib38] Hsiao HW, Tsai KL, Wang LF, Chen YH, Chiang PC, Chuang SM et al. The decline of autophagy contributes to proximal tubular dysfunction during sepsis. Shock 2017; 8: 289–29637. 10.1097/SHK.0b013e318240b52a 22089196

[bib39] Tu Y, Gu L, Chen D, Wu W, Liu H, Hu H et al. Rhein inhibits autophagy in rat renal tubular cells by regulation of AMPK/mTOR signaling. Sci Rep 2017; 7: 43790. 28252052 10.1038/srep43790 PMC5333140

[bib40] Song XB, Liu G, Liu F, Yan ZG, Wang ZY, Liu ZP et al. Autophagy blockade and lysosomal membrane permeabilization contribute to lead-induced nephrotoxicity in primary rat proximal tubular cells. Cell Death Dis 2017; 8: e2863. 28594408 10.1038/cddis.2017.262 PMC5520918

[bib41] Zhao C, Chen Z, Qi J, Duan S, Huang Z, Zhang C et al. Drp1-dependent mitophagy protects against cisplatin-induced apoptosis of renal tubular epithelial cells by improving mitochondrial function. Oncotarget 2017; 8: 20988–21000. 28423497 10.18632/oncotarget.15470 PMC5400560

[bib42] Richards AB, Krakowka S, Dexter LB, Schmid H, Wolterbeek AP, Waalkens-Berendsen DH et al. Trehalose: a review of properties, history of use and human tolerance, and results of multiple safety studies. Food Chem Toxicol 2002; 40: 871–898. 12065209 10.1016/s0278-6915(02)00011-x

[bib43] Matsuo T, Tsuchida Y, Morimoto N. Trehalose eye drops in the treatment of dry eye syndrome. Ophthalmology 2002; 109: 2024–2029. 12414409 10.1016/s0161-6420(02)01219-8

[bib44] Wang L, Lin SQ, He YL, Liu G, Wang ZY. Protective effects of quercetin on cadmium-induced cytotoxicity in primary cultures of rat proximal tubular cells. Biomed Environ Sci 2013; 26: 258–267. 23534466 10.3967/0895-3988.2013.04.004

[bib45] Klionsky DJ, Abdelmohsen K, Abe A, Abedin MJ, Abeliovich H, Acevedo Arozena A et al. Guidelines for the use and interpretation of assays for monitoring autophagy (3rd edition). Autophagy 2016; 12: 1–222. 26799652 10.1080/15548627.2015.1100356 PMC4835977

[bib46] Jiang P, Mizushima N. LC3- and p62-based biochemical methods for the analysis of autophagy progression in mammalian cells. Methods 2015; 75: 13–18. 25484342 10.1016/j.ymeth.2014.11.021

[bib47] Lee WK, Probst S, Santoyo-Sánchez MP, Al-Hamdani W, Diebels I, von Sivers JK et al. Initial autophagic protection switches to disruption of autophagic flux by lysosomal instability during cadmium stress accrual in renal NRK-52E cells. Arch Toxicol 2017; 91: 3225–3245. 28321485 10.1007/s00204-017-1942-9

[bib48] Eisenberg-Lerner A, Kimchi A. PKD is a kinase of Vps34 that mediates ROS-induced autophagy downstream of DAPk. Cell Death Differ 2012; 19: 788–797. 22095288 10.1038/cdd.2011.149 PMC3321617

[bib49] Choi J, Jo M, Lee E, Choi D. Induction of apoptotic cell death via accumulation of autophagosomes in rat granulosa cells. Fertil Steril 2011; 95: 1482–1486. 20630503 10.1016/j.fertnstert.2010.06.006

[bib50] Liu L, Zhang N, Dou Y, Mao G, Bi C, Pang W et al. Lysosomal dysfunction and autophagy blockade contribute to IMB-6G-induced apoptosis in pancreatic cancer cells. Sci Rep 2017; 7: 41862. 28139733 10.1038/srep41862 PMC5282566

[bib51] Chen S, Yuan J, Yao S, Jin Y, Chen G, Tian W et al. Lipopolysaccharides may aggravate apoptosis through accumulation of autophagosomes in alveolar macrophages of human silicosis. Autophagy 2015; 11: 2346–2357. 26553601 10.1080/15548627.2015.1109765 PMC4835201

[bib52] Zhou J, Tan SH, Nicolas V, Bauvy C, Yang ND, Zhang J et al. Activation of lysosomal function in the course of autophagy via mTORC1 suppression and autophagosome–lysosome fusion. Cell Res 2013; 23: 508–523. 23337583 10.1038/cr.2013.11 PMC3616426

[bib53] Hubert V, Peschel A, Langer B, Gröger M, Rees A, Kain R. LAMP-2 is required for incorporating syntaxin-17 into autophagosomes and for their fusion with lysosomes. Biol Open 2016; 5: 1516–1529. 27628032 10.1242/bio.018648 PMC5087675

[bib54] Rong Y, McPhee CK, Deng S, Huang L, Chen L, Liu M et al. Spinster is required for autophagic lysosome reformation and mTOR reactivation following starvation. Proc Natl Acad Sci USA 2011; 108: 7826–7831. 21518918 10.1073/pnas.1013800108 PMC3093520

[bib55] Gerónimo-Olvera C, Montiel T, Rincon-Heredia R, Castro-Obregón S, Massieu L. Autophagy fails to prevent glucose deprivation/glucose reintroduction-induced neuronal death due to calpain-mediated lysosomal dysfunction in cortical neurons. Cell Death Dis 2017; 8: e2911. 28661473 10.1038/cddis.2017.299 PMC5520945

[bib56] Kaminskyy V, Zhivotovsky B. Proteases in autophagy. Biochim Biophys Acta 2012; 1824: 44–50. 21640203 10.1016/j.bbapap.2011.05.013

[bib57] Turk B, Turk D, Turk V. Lysosomal cysteine proteases: more than scavengers. Biochim Biophys Acta 2000; 1477: 98–111. 10708852 10.1016/s0167-4838(99)00263-0

[bib58] Orr ME, Oddo S. Autophagic/lysosomal dysfunction in Alzheimer’s disease. Alzheimers Res Ther 2013; 5: 1–9. 24171818 10.1186/alzrt217 PMC3979020

[bib59] Rodríguez-Navarro JA, Rodríguez L, Casarejos MJ, Solano RM, Gómez A, Perucho J et al. Trehalose ameliorates dopaminergic and tau pathology in parkin deleted/tau overexpressing mice through autophagy activation. Neurobiol Dis 2010; 39: 423–438. 20546895 10.1016/j.nbd.2010.05.014

[bib60] Schaeffer V, Lavenir I, Ozcelik S, Tolnay M, Winkler DT, Goedert M. Stimulation of autophagy reduces neurodegeneration in a mouse model of human tauopathy. Brain 2012; 135: 2169–2177. 22689910 10.1093/brain/aws143 PMC3381726

[bib61] Castillo K, Nassif M, Valenzuela V, Rojas F, Matus S, Mercado G et al. Trehalose delays the progression of amyotrophic lateral sclerosis by enhancing autophagy in motoneurons. Autophagy 2013; 9: 1308–1320. 23851366 10.4161/auto.25188

[bib62] Liu G, Wang ZK, Wang ZY, Yang DB, Liu ZP, Wang L. Mitochondrial permeability transition and its regulatory components are implicated in apoptosis of primary cultures of rat proximal tubular cells exposed to lead. Arch Toxicol 2016; 90: 1193–1209. 26082307 10.1007/s00204-015-1547-0

[bib63] Wang L, Wang H, Hu M, Cao J, Chen D, Liu ZP. Oxidative stress and apoptotic changes in primary cultures of rat proximal tubular cells exposed to lead. Arch Toxicol 2009; 83: 417–427. 19347332 10.1007/s00204-009-0425-z

[bib64] Bolte S, Cordelieres FP. A guided tour into subcellular colocalization analysis in light microscopy. J Microsc 2006; 224: 213–232. 17210054 10.1111/j.1365-2818.2006.01706.x

